# Sex- and Age-Related Differences in Physiological [^18^F]FDOPA Uptake on Long Axial Field-of-View PET/CT Imaging

**DOI:** 10.3390/bioengineering13060700

**Published:** 2026-06-18

**Authors:** Tara M. Tabak, Joyce van Sluis, Floris H. P. van Velden, Lioe-Fee. F. de Geus-Oei, Françoise J. Siepel, Riemer H. J. A. Slart

**Affiliations:** 1 Faculty of Science and Technology, University of Twente, 7522 NB Enschede, The Netherlands; l.f.de_geus-oei@lumc.nl (L.-F.F.d.G.-O.); f.j.siepel@utwente.nl (F.J.S.); r.h.j.a.slart@umcg.nl (R.H.J.A.S.); 2 Department of Nuclear Medicine and Molecular Imaging, University Medical Center Groningen, 9713 GZ Groningen, The Netherlands; j.van.sluis@umcg.nl; 3 Department of Radiology, Section of Nuclear Medicine, Leiden University Medical Center, 2333 ZA Leiden, The Netherlands; f.h.p.van_velden@lumc.nl; 4 Department of Radiation Science & Technology, Delft University of Technology, 2629 JB Delft, The Netherlands

**Keywords:** [^18^F]FDOPA, PET/CT, age, biodistribution, sex

## Abstract

This retrospective quantitative data analysis study aimed to investigate sex- and age-related differences in the physiological distribution of [^18^F]FDOPA uptake in long axial field-of-view (LAFOV) PET images across a range of organs and tissues. A retrospective quantitative data analysis study of 106 anonymized PET/CT images acquired from vertex to mid-thigh with minimal abnormalities, divided in two gender groups and two age groups was used for this study. The mean and max lean body mass weighted standardized uptake values ($SUL_{mean}$, $SUL_{max}$), target-to-background ratios (TBR), and coefficients of variation (CoV) were used to quantify tracer uptake. Sex- and age-related differences in uptake were organ- and metric-specific. Most organs showed comparable uptake between males and females. However, males exhibited higher absolute uptake in metabolically active organs and females showed greater intra-organ heterogeneity. Aging was generally associated with increased tracer uptake and variability, especially in women, with the hip showing higher uptake in younger individuals. Statistically significant differences were most prominent in women and varied by organ and metric. In conclusion, both sex and age significantly influence [^18^F]FDOPA PET tracer uptake and variability in an organ- and metric-specific manner. Incorporating sex- and age-adjusted reference values may improve the accuracy and personalization of PET imaging in clinical and research settings.

## 1. Introduction

[^18^F]FDOPA is a radiolabeled amino acid analogue of L-DOPA and serves as a marker of dopamine synthesis [1,2]. Via the large amino acid transporter system, [^18^F]FDOPA follows the dopaminergic metabolic pathway and is decarboxylated to form fluorodopamine. This mechanism allows [^18^F]FDOPA positron emission tomography (PET) to visualize and quantify the integrity and activity of dopaminergic systems in the brain as well as in peripheral tissues.

Premedication with carbidopa increases plasma [^18^F]FDOPA availability and reduces renal excretion, resulting in improved image quality [1]. However, carbidopa premedication is not recommended for the evaluation of pancreatic lesions [1].

[^18^F]FDOPA PET is commonly used to assess the integrity of the striatal dopaminergic system in patients with Parkinson’s disease [3]. In addition to neurodegenerative imaging, [^18^F]FDOPA PET is applied in oncological and endocrinological settings, including the evaluation of neuroendocrine and brain tumors, and pancreatic cell hyperplasia where increased [^18^F]FDOPA uptake is typically observed [1].

Physiological [^18^F]FDOPA uptake is observed in the basal ganglia, pancreas, liver, spleen, and salivary glands. Activity in the gallbladder, bile ducts, and urinary tract predominantly reflects tracer excretion [1,3].

Although the physiological biodistribution of [^18^F]FDOPA is well described, potential differences between male and female patients remain not fully understood. This is notable given the evidence for sex-related differences in the dopamine system [4,5]. Studies have shown that women exhibit higher baseline dopaminergic activity than men, particularly in the striatum, along with faster dopamine synthesis, uptake, and release. These biological differences are reflected in the prevalence, symptomatology, and treatment response of dopamine-related neuropsychiatric disorders [5]. Consequently, systematic investigations into male–female differences in physiological [^18^F]FDOPA uptake across organs and tissues are limited, highlighting an important gap in the current understanding of [^18^F]FDOPA PET biodistribution.

This report addresses the central research question: What are the differences in the physiological activity distribution between male and female [^18^F]FDOPA PET scans? A secondary objective is to assess the influence of age on [^18^F]FDOPA uptake, with patients categorized into groups of 18–50 years and 51–90 years. Physiological uptake is analyzed using the mean and maximum standardized uptake value normalized to lean body mass (SUL), target-to-background ratio (TBR), and the coefficient of variation (CoV). SUL is used instead of the body-weighted SUV to reduce bias related to body composition differences between men and women [6]. The CoV provides additional information on intra-organ heterogeneity. The organs and tissues evaluated include the adrenal glands, bone marrow in the hip bones, brain, duodenum, gallbladder, kidneys, left ventricle, liver, pancreas, spleen, stomach, and thyroid [1].

By quantitatively assessing sex- and age-related differences in [^18^F]FDOPA biodistribution, this study aims to improve the understanding of physiological variability in [^18^F]FDOPA PET imaging and to support more accurate interpretation in both clinical and research settings.

## 2. Materials and Methods

In this retrospective quantitative data analysis study, [^18^F]FDOPA uptake was quantified using the $SUL_{mean}$, $SUL_{max}$, TBR, and CoV. Here, $SUL_{mean}$ is the SUL of the mean activity in the volume of interest, and $SUL_{max}$ is the SUL of the maximal activity in the volume of interest. These metrics allow analysis based on activity per organ or tissue in comparison to the background, which is taken as the aorta in this study. The CoV will give information about the heterogeneity within an organ or tissue. The following organs and tissues were included: adrenal glands, hip bones as the bone marrow, brain, duodenum, gallbladder, kidneys, left ventricle, liver, pancreas, spleen, stomach, and thyroid [1,7]. Comparisons are made between male and female and age groups 18–50 and 51–90 as an indirect proxy for menopausal status.

### 2.1. Materials

**Figure 1:**
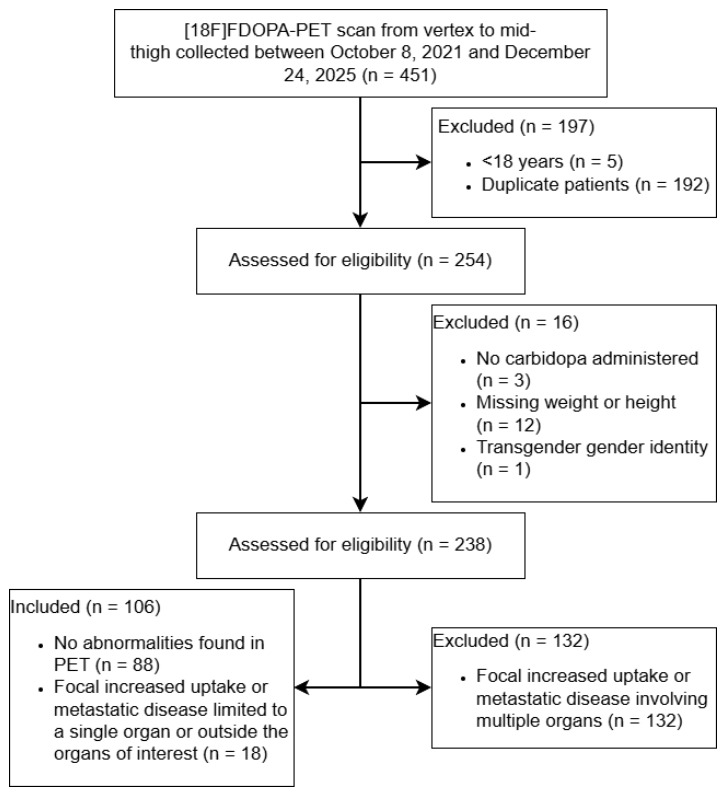
A STROBE flow diagram illustrating the selection process of patients who underwent [^18^F]FDOPA PET/CT between 2021 and 2025. Eligibility criteria, exclusions, and the final analytical sample are displayed.

A dataset of 106 anonymized [^18^F]FDOPA PET and low-dose CT images acquired from vertex to mid-thigh, performed on the Biograph Vision Quadra was used for this study [8]. The data were collected between 8 October 2021 and 24 December 2025 at the University Medical Center Groningen (UMCG). Patients were required to be between 18 and 90 years of age and to have received carbidopa prior to the scan [1]. Additionally, weight, height, and administered [^18^F]FDOPA activity had to be available. Only patients with no abnormalities or focal increased uptake or metastatic disease limited to a single organ or outside the organs of interest on PET were included. A complete overview of the patient selection process can be found in Figure 1. In the case of multiple scans, in compliance with the inclusion criteria, the first baseline scan will be included.

The clinical reports in the electronic patient dossier (EPD) (version Hyperspace Augustus 2025; EPIC, Verona, WI, USA) and the imaging software Syngo.via (version Enterprise Browser VB80G; Siemens Healthineers, Forchheim, Germany) were used to assess eligibility and anonymize the dataset. For automatic segmentation of the organs and tissues the segmentation model MOOSE (version 3.0; CLARITY, Gleisdorf, Austria) was used [9,10]. Collecting and processing the segmentation results into the outcome values was done using MATLAB (version R2024b; The MathWorks, Natick, MA, USA). Throughout the previous steps Excel (version 2603; Microsoft Office, Redmond, WA, USA) was used to collect data and check results. The statistical analysis was performed using SPSS Statistics (version 30.0.0.0; IBM, Armonk, NY, USA).

### 2.2. Study Design

For consistent precision and comparison with previous gender difference studies in the Nuclear Medicine and Molecular Imaging department of the UMCG—where a confidence interval of 92% and a margin of error of 10% are used—the sample size was chosen as similar. Due to the hormonal essence of [^18^F]FDOPA, the hypothesis is that females show a shift in physiologic activity after menopause. To account for hormonal differences, two age groups were formed: aged 18–50 years and 51–90 years. The total sample size of 106 patients, divided in two gender groups and two age groups, was used. No formal a priori sample size calculation was performed, as this retrospective study is exploratory in nature and aimed at generating hypotheses rather than confirming predefined effect sizes.

For each patient, the results of the [^18^F]FDOPA PET/CT were collected using the official report via EPIC EPD. This includes the administered [^18^F]FDOPA activity and carbidopa dose, body weight, height and abnormalities in scan or anatomy. The PET data were acquired using the long axial field-of-view (LAFOV) Biograph Vision Quadra PET/CT (Siemens Healthineers, Germany).

All PET data were fully corrected and reconstructed using an iterative reconstruction algorithm, including resolution modelling, with 4 iterations and 5 subsets, a matrix size of 220 × 220 and Gaussian filtering of 5 mm full width at half maximum (FWHM) in accordance with the European Association of Nuclear Medicine (EANM) Research Ltd. F18 standards 2 (EARL2) accreditation program [11,12]. Both the low-dose CT and PET scans were anonymized using Syngo.via and exported for segmentation. MOOSE was used to segment the adrenal glands, brain, duodenum, gallbladder, hip, kidneys, left ventricle, liver, pancreas, spleen, stomach, and thyroid. Some organs and tissues consist of multiple parts, like the left and right kidney. These were taken into account separately. For segmentation, the low-dose CT images were used. The MOOSE models used for segmentations are clin_ct_cardiac, clin_ct_digestive, clin_ct_organs, clin_ct_peripheral_bones and clin_ct_vertebrae [9,10]. The segmentation results are for each organ or tissue the volume in ${\mathrm{mm}}^{3}$ and the PET activity in kBq/mL including the mean, maximum, minimum, median and standard deviation.

### 2.3. Data Analysis

The results from the segmentation were collected and processed using MATLAB R2024b and Microsoft Excel. To calculate the SUL, TBR and CoV the following Formulas (1)–(5) were used, and the results exported to Microsoft Excel [6,13,14,15].

(1) $$SUL=\frac{Act_{VOI}\;\left[\mathrm{MBq}/\mathrm{mL}\right]}{Act_{administered}\;\left[\mathrm{MBq}\right]/\mathrm{LBM}\left[\mathrm{kg}\right]}$$

(2) $$LBM_{man}=\frac{9270\cdot Total\;body\;weight\;[\mathrm{kg}]}{6680+(216\cdot \left(\frac{Total\;body\;weight\;[\mathrm{kg}]}{{\left(Height\;\left[m\right]\right)}^{2}}\right))}$$

(3) $$LBM_{vrouw}=\frac{9270\cdot Total\;body\;weight\;[\mathrm{kg}]}{8780+(244\cdot \left(\frac{Total\;body\;weight\;[\mathrm{kg}]}{{\left(Height\;\left[m\right]\right)}^{2}}\right))}$$

(4) $$TBR=\frac{SUL_{target}}{SUL_{background}}$$

(5) $$CoV=\frac{SD_{mean}}{SUL_{mean}}$$

where $Act_{VOI}$ is the mean or maximum radioactivity concentration in the organ or tissue, $Act_{administered}$ is the administered dose at t=0, $SUL_{background}$ is the $SUL_{mean}$ of the aorta, and $SD_{mean}$ is the standard deviation of the $Act_{VOI}$ in the organ or tissue.

These metrics were visualized using boxplots with the median and the interquartile range (IQR) to identify outliers. An extreme outlier was defined as a value exceeding three times the interquartile range (IQR) above the third quartile or below the first quartile. Outliers were evaluated for pathology and segmentation errors to determine the need for exclusion of that metric or all metrics.

### 2.4. Statistical Analysis

For each organ or tissue, $SUL_{mean}$, $SUL_{max}$, TBR and CoV were compared and tested for statistically significant differences between sex and age groups using SPSS Statistics. Age differences are determined within gender groups to eliminate sex-based differences. The following comparisons are made.

1. Comparison between all included males and females;
2. Comparison between males age 18–50 and males age 51–90;
3. Comparison between females age 18–50 and females age 51–90.

Organ and outcome PET metrics per gender and age group were separately evaluated for normalcy using the Shapiro–Wilk test [16]. A *p*-value below 0.05 was considered significant, meaning normality cannot be assumed and the group is considered non-parametric. Depending on the results of the Shapiro–Wilk test either a parametric independent *t*-test or a non-parametric Mann–Whitney U test was performed [17,18,19]. Here the *p*-value of p<0.05 was used as well. In both the parametric and the non-parametric test the two-tailed p value was taken.

This study includes multiple statistical tests using the same data set, which increases the risk of a type I error [20]. However, given the exploratory nature of this research and its focus on identifying potential differences in physiological [^18^F]FDOPA uptake, reducing type II error was prioritized. Therefore, no formal correction for multiple comparisons was applied.

## 3. Results

An overview of the characteristics of the included patients is shown in Table 1. In some patients organs or tissues were resected or pathologies were found. These organs or tissues were removed from the study. In Table 2 the excluded organs including reason are listed.

### 3.1. Comparison Between Male and Female

One extreme outlier in the $SUL_{max}$ of the right kidney was identified within the male group, with a value exceeding three times the interquartile range (IQR) above the third quartile and approximately 15 times the group mean. Therefore, all outcome values for this organ were removed from the dataset. The number of cases per organ is presented in Table 3, and this count is consistent across all metrics.

**Figure 2:**
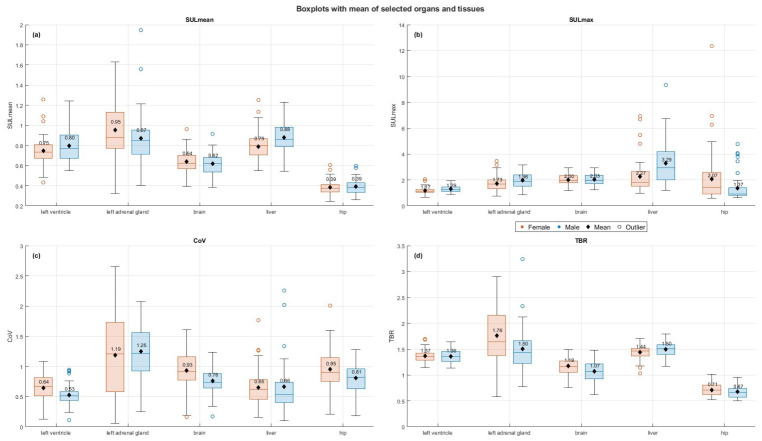
Boxplots with mean of the (**a**) $SUL_{mean}$, (**b**) $SUL_{max}$, (c) TBR and (**d**) CoV of selected organs with a minimum of two parameters with statistically significant sex-differences. Please note that the axes are intentionally scaled differently to improve visualization.

Boxplots illustrating the metrics for each organ can be found in Figure 2 and Appendix A. For each outcome variable, normality was assessed, and based on these results, appropriate statistical tests were applied for every metric and organ or tissue. Table 4 displays the results of the Shapiro–Wilk test. For variables meeting normality assumptions, the independent samples *t*-test was used and for non-parametric data the Mann–Whitney U test was employed. Detailed results are provided in Appendix A.

#### 3.1.1. $SUL_{mean}$ in Sex Comparison

The independent samples *t*-test revealed no statistically significant sex differences in the $SUL_{mean}$ for the duodenum, brain, pancreas, stomach, or thyroid. Similarly, the Mann–Whitney U test indicated no significant differences between sexes for the $SUL_{mean}$ in the left ventricle, adrenal glands, gallbladder, spleen, or hip. However, statistically significant sex differences were observed in the $SUL_{mean}$ of the kidneys and the liver. Specifically, males exhibited a higher $SUL_{mean}$ in the left kidney (M=1.603;SD=0.773) versus females (M=1.351;SD=0.288), in the right kidney (M=1.542;SD=0.285) versus females (M=1.449;SD=0.356) and in the liver (M=0.881;SD=0.152) versus females (M=0.789;SD=0.141). In all statistically significant cases, males showed higher mean $SUL_{mean}$ values than females.

#### 3.1.2. $SUL_{max}$ in Sex Comparison

No statistically significant sex differences were observed for the $SUL_{max}$ in the right adrenal gland, brain, gallbladder, kidneys, spleen, or stomach. However, the Mann–Whitney U test indicated statistically significant sex difference in the left ventricle, were males (M=1.293;SD=0.245) had a higher $SUL_{max}$ than female (M=1.169;SD=0.242), and in the duodenum with a higher $SUL_{max}$ in males (M=1.929;SD=1.460) than females (M=1.591;SD=1.232). The left adrenal gland also showed higher $SUL_{max}$ in males (M=1.964;SD=0.581) versus females (M=1.734;SD=0.563), along with the liver (M=3.294;SD=1.781) versus females (M=2.272;SD=1.252) and the pancreas (M=2.051;SD=0.682) versus females (M=1.670;SD=0.588). The thyroid had a higher $SUL_{max}$ in males (M=0.709;SD=0.115) than females (M=0.646;SD=0.115), whereas the hip demonstrated a higher $SUL_{max}$ in females (M=2.068;SD=1.928) compared to males (M=1.369;SD=1.007). In all statistically significant cases, except for the hip, males had higher $SUL_{max}$ values than females.

#### 3.1.3. TBR in Sex Comparison

No statistically significant sex differences were detected for TBR in the left ventricle, duodenum, right adrenal gland, gallbladder, kidneys, pancreas, spleen, stomach, thyroid, or hip. Significant differences were found for the brain, with females (M=1.179;SD=0.154) having higher TBR than males (M=1.072;SD=0.199), along with the left adrenal gland (M=1.760;SD=0.494) versus males (M=1.503;SD=0.427). For the liver males (M=1.497;SD=0.125) show a higher TBR versus females (M=1.441;SD=0.133).

#### 3.1.4. CoV in Sex Comparison

No statistically significant sex differences were found in the duodenum, adrenal glands, gallbladder, left kidney, liver, pancreas, or thyroid. The independent samples *t*-test identified statistically significant sex difference in the brain, where females (M=0.930;SD=0.306) had a higher CoV than males (M=0.764;SD=0.223), and the hip (M=0.950;SD=0.325) versus males (M=0.808;SD=0.230). The Mann–Whitney U test revealed higher CoV in females in the left ventricle (M=0.644;SD=0.228) versus males (M=0.527;SD=0.169), right kidney (M=1.189;SD=0.612) versus males (M=0.999;SD=0.522), stomach (M=1.894;SD=1.020) versus males (M=1.471;SD=0.644), and spleen (M=0.899;SD=0.518) versus males (M=0.672;SD=0.375). In all statistically significant cases, females exhibited higher mean CoV values than males.

### 3.2. Comparison Between Age

An extreme outlier in $SUL_{max}$ for the right kidney was found in the male group aged 51–90 years, all outcome values for this organ were removed from the dataset. The number of cases per organ for the two age groups is listed in Table 5, and this count is consistent across all metrics.

**Figure 3:**
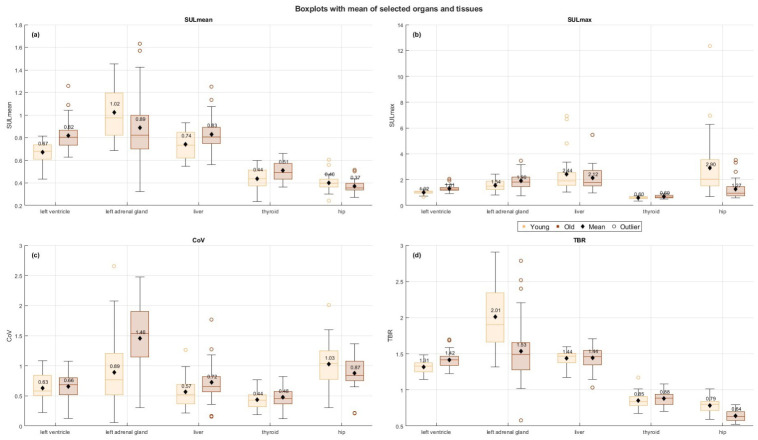
Boxplots with mean of the (**a**) $SUL_{mean}$, (**b**) $SUL_{max}$, (**c**) TBR and (**d**) CoV of selected organs with a minimum of two parameters with statistically significant age-differences in females. Please note, the axes are intentionally scaled differently to improve visualization.

**Figure 4:**
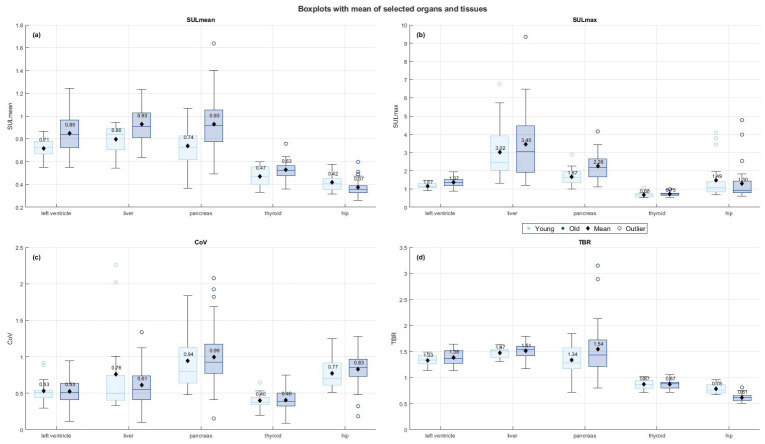
Boxplots with mean of the (**a**) $SUL_{mean}$, (**b**) $SUL_{max}$, (**c**) TBR and (**d**) CoV of selected organs with a minimum of two parameters with statistically significant age-differences in males. Please note, the axes are intentionally scaled differently to improve visualization.

Boxplots for the metrics by organ are shown in Figure 3 for female age comparison and Figure 4 for male age comparison, as well as in Appendix A. For each outcome, normality was calculated, and appropriate statistical tests were performed. Table 6 presents the results of the normality test. The independent samples *t*-test was used for parametric groups, and the Mann–Whitney U test for non-parametric groups. Complete test results are included in Appendix A.

#### 3.2.1. $SUL_{mean}$ in Age Comparison

No statistically significant age differences were detected in the right adrenal gland, brain, gallbladder, kidneys, and spleen for either sex. Likewise, no significant age difference was found in the left adrenal gland in males and the pancreas in females.

In females, significant age differences were found with older women having higher $SUL_{mean}$ in the left ventricle (M=0.819;SD=0.130) versus (M=0.671;SD=0.096), duodenum (M=0.671;SD=0.109) versus (M=0.602;SD=0.124), liver (M=0.831; SD=0.147) versus (M=0.742;SD=0.118), stomach (M=0.447;SD=0.122) versus (M=0.352;SD=0.132), and thyroid (M=0.508;SD=0.080) versus (M=0.438; SD=0.088). The $SUL_{mean}$ for the left adrenal gland and hip was higher in younger females (M=1.025;SD=0.216) versus (M=0.889;SD=0.292) and (M=0.401;SD=0.072) versus (M=0.370;SD=0.053) respectively.

For males, older individuals showed higher $SUL_{mean}$ in the left ventricle (M=0.848; SD=0.171) versus (M=0.715;SD=0.080), duodenum (M=0.697;SD=0.156) versus (M=0.593;SD=0.128), liver (M=0.929;SD=0.150) versus (M=0.798; SD=0.115), pancreas (M=0.927;SD=0.250) versus (M=0.736;SD=0.181), stomach (M=0.452;SD=0.138) versus (M=0.368;SD=0.127), and thyroid (M=0.528; SD=0.076) versus (M=0.468;SD=0.079). The hip $SUL_{mean}$ was higher in younger males (M=0.418;SD=0.067) versus (M=0.374;SD=0.074).

#### 3.2.2. $SUL_{max}$ in Age Comparison

No statistically significant age differences were found in the duodenum, right adrenal gland, gallbladder, kidneys, liver, and stomach in males and females. Moreover, no significant age difference was detected in the left adrenal gland, brain, thyroid and hip for males, or in the pancreas for females.

Among females, older age groups showed higher $SUL_{max}$ in the left ventricle (M=1.310;SD=0.238) versus (M=1.023;SD=0.136), left adrenal gland (M=1.903;SD=0.636) versus (M=1.545;SD=0.388), brain (M=2.135;SD=0.309) versus (M=1.845;SD=0.325), thyroid (M=0.686;SD=0.109) versus (M=0.602; SD=0.104), and hip (M=1.269;SD=0.752) versus (M=2.895;SD=2.375).

In males, significant age differences were observed only in the left ventricle and pancreas. In both cases, older males had higher $SUL_{max}$ values. In the left ventricle, (M=1.369;SD=0.261) versus (M=1.168;SD=0.148), and pancreas (M=2.263; SD=0.689) versus (M=1.667;SD=0.469).

In all statistically significant cases, higher $SUL_{max}$ values were found in older groups for both sexes.

#### 3.2.3. TBR in Age Comparison

No statistically significant age differences were found in the duodenum, gallbladder, liver, stomach, and thyroid. Likewise, no significant age difference was found in the left ventricle, left adrenal gland, kidneys, and pancreas in males and in the brain in females.

In females, the TBR of the left ventricle was higher compared in older women (M=1.417;SD=0.111) compared to younger women (M=1.314;SD=0.088). The younger age groups showed a higher TBR in the left adrenal gland (M=2.011;SD=0.407) versus (M=1.534;SD=0.455), right adrenal gland (M=1.836;SD=0.570) versus (M=1.480;SD=0.352), left kidney (M=2.660;SD=0.504) versus (M=2.329; SD=0.317), right kidney (M=2.452;SD=0.358) versus (M=2.430;SD=0.258), pancreas (M=1.680;SD=0.327) versus (M=1.389;SD=0.203), spleen (M=1.057; SD=0.072) versus (M=0.961;SD=0.163), and hip (M=0.786;SD=0.088) versus (M=0.642;SD=0.075).

Similarly, in males, younger men demonstrated higher TBR in the right adrenal gland (M=1.636;SD=0.533) versus (M=1.401;SD=0.330), brain (M=1.194;SD=0.137) versus (M=0.999;SD=0.195), spleen (M=1.112;SD=0.075) versus (M=1.062; SD=0.075), and hip (M=0.780;SD=0.084) versus (M=0.613;SD=0.071).

For the majority of organs with statistically significant age differences, higher TBR values were observed in the younger age groups for both males and females, except for the left ventricle in females, where older women exhibited higher TBR.

#### 3.2.4. CoV in Age Comparison

No significant age differences in CoV were found in males. In females, no significant differences were observed in the left ventricle, duodenum, brain, gallbladder, kidneys, spleen, or hip. Significant age differences were found in the adrenal glands, liver, pancreas, and stomach. In the stomach, younger females (M=2.227;SD=1.117) had a higher CoV than older females (M=1.535;SD=0.754). Older females exhibited higher CoV in the left adrenal gland (M=1.456;SD=0.588) versus (M=0.893;SD=0.600), right adrenal gland (M=1.371;SD=0.600) versus (M=0.975;SD=0.655), liver (M=0.723; SD=0.326) versus (M=0.566;SD=0.251), and pancreas (M=1.102;SD=0.567) versus (M=0.792;SD=0.300).

## 4. Discussion

This study examined sex-related differences in multiple PET-derived parameters across several organs. Overall, the findings suggest that statistically significant sex differences are organ-specific, with most organs showing comparable values between males and females.

For $SUL_{mean}$, no statistically significant sex differences were observed in the majority of organs, suggesting similar average tracer uptake. In the kidneys and liver, however, males exhibited a statistically significant higher $SUL_{mean}$. While the observed differences could be associated with variations in metabolic activity, organ size, body composition, lean body mass, or organ perfusion, causal relationships cannot be determined from this study. Moreover, the influence of other confounding factors, such as age, BMI, hormonal status, or medication use cannot be ruled out. Future studies are needed to clarify the underlying causes.

Statistically significant sex differences were observed more frequently for $SUL_{max}$. Males generally demonstrated higher $SUL_{max}$ values in several organs, including the left ventricle, duodenum, left adrenal gland, liver, pancreas, and thyroid, while females showed higher $SUL_{max}$ values in the hip. While these findings suggest that maximum uptake may be more sensitive to sex-related physiological differences, it is important to recognize that $SUL_{max}$ is especially sensitive to focal uptake, local variability, noise, and outliers [21]. This is relevant given that the sample sizes for some subgroups may limit the robustness of these findings and increase susceptibility to statistical fluctuation.

For TBR, most organs did not exhibit significant sex differences, indicating that the target-to-background ratio is largely comparable between sexes. Since TBR normalizes uptake using the blood pool SUL, it likely reduces the magnitude of inter-individual and inter-groups variation. Nevertheless, organ-specific differences were observed, with females showing higher TBR in the brain and left adrenal gland, and males exhibiting higher TBR in the liver.

Analysis of the CoV revealed that females consistently exhibited greater tracer uptake variability in all organs with statistically significant sex differences, including the brain, hip, left ventricle, right kidney, and spleen. Increased intra-organ heterogeneity in females may be influenced by hormonal fluctuations, including the menstrual cycle, anatomical diversity or other physiological factors. This elevated variability is clinically relevant because it may compromise the reliability and repeatability of quantitative PET measurements capturing uptake heterogeneity. Such effects are particularly important when organs with pronounced physiological heterogeneity are used as reference values, as high intra-organ variability may reduce both the sensitivity and specificity of PET-based diagnostics or therapy response monitoring in women.

Overall, while males tend to demonstrate higher absolute tracer uptake, particularly in metabolically active organs such as the liver and kidneys, females show greater uptake heterogeneity across several organs. Notably, the liver, which is frequently used as a reference organ in 2-deoxy-2-[^18^F]fluoro-D-glucose ([^18^F]FDG) PET studies, consistently showed statistically significant higher uptake in males across multiple parameters. The use of a universal or non-sex-specific reference threshold or normalization approach might introduce bias in both clinical and research PET studies. Consideration of sex-adjusted thresholds or normalization techniques may be warranted to ensure robust and unbiased quantitative PET interpretation.

Furthermore, this study examined age-related differences in multiple PET-derived parameters across several organs, with results indicating that the presence, direction, and magnitude of age effects are organ- and metric-specific.

For the $SUL_{mean}$, statistically significant age differences were found more frequently in women than in men. In both sexes, most organs with significant differences were the same and showed the same direction of difference. Older individuals generally exhibited higher $SUL_{mean}$ values in the left ventricle, duodenum, liver, stomach, and thyroid, while the hip showed higher $SUL_{mean}$ values in younger groups, contrasting with other organs where $SUL_{mean}$ increased with age. This pattern may reflect distinct age-related changes in skeletal metabolism compared to soft tissues [22].

For the $SUL_{max}$, statistically significant age differences were observed far more often in women than in men. In men, only the left ventricle and pancreas showed higher $SUL_{max}$ in older groups. In women, several organs showed significant age-related increases in $SUL_{max}$, such as the left ventricle, left adrenal gland, brain, thyroid, and hip. In all cases, $SUL_{max}$ increased with age. As $SUL_{max}$ represents maximum uptake, these findings suggest that aging is associated with increased focal tracer accumulation in certain tissues—an effect that is more pronounced in women. However, as with the sex analysis, it must be noted that $SUL_{max}$ is particularly sensitive to focal uptake, outliers, and noise, and may therefore be less robust, especially in smaller subgroups or tissues with high variability.

For TBR, which normalized uptake to the reference region, the majority of organs showed no significant age differences, particularly in men. In women, however, more statistically significant differences were found, especially in the adrenal glands, spleen, and hip. In most organs with significant differences, younger individuals had higher TBR values, but an exception was the left ventricle in females, where older women had higher TBR. The more frequent age differences in women may reflect hormonal changes with aging, while normalization with TBR reduces but does not completely eliminate age-related and organ-specific impacts.

The CoV revealed no significant age effects in men, with CoV values remaining relatively stable across age groups. In females, though, the adrenal glands, liver, pancreas, and stomach displayed statistically significantly higher CoV in older individuals, whereas in the stomach, younger females showed greater variability. These findings indicate that increased intra-organ heterogeneity with age is predominantly a phenomenon observed in women. This might be linked to hormonal, anatomical, or physiological changes due to the transition through menopause. As a result, higher CoV in female organs with aging could compromise the reliability and repeatability of quantitative PET analysis involving measures that capture uptake heterogeneity, especially if such organs are used for reference or normalization.

These findings suggest that aging is associated with increased tracer uptake and heterogeneity in several organs, with the effects typically being larger and more widespread in women than in men. Notably, while virtually all organs with statistically significant age differences experienced increased average and maximum uptake with age, the hip showed a distinctive pattern with higher values in younger individuals. This underscores the complexity of age-related metabolic changes across tissues.

The observation that more significant age differences were found in women might relate to hormonal transitions, such as menopause, influencing metabolism in multiple organs [23]. These findings must be interpreted with caution, since causal relationships cannot be determined from this study due to unmeasured confounders such as BMI, comorbidities, medication use, or precise menopausal status and hormonal profile. Furthermore, some findings may result from technical or sampling variability, especially for metrics such as $SUL_{max}$ and CoV that are sensitive to outliers, local hotspots and intraregional heterogeneity.

Finally, several limitations must be acknowledged. This study was not designed to identify the underlying mechanisms of observed sex or age differences. Confounding factors such as hormonal status, menopausal phase, co-morbidities, and medication use were not fully controlled for and could have contributed to the observed patterns. In addition, the sample sizes for organ-specific subgroups were limited, potentially reducing statistical power and the stability of parameters especially sensitive to outliers, such as $SUL_{max}$ and CoV. Furthermore, no correction for multiple comparisons was applied given the exploratory nature of this study, where minimizing type II error and identifying potential signals was prioritized over limiting false positives. As a result, the risk of type I error is increased, and findings should be interpreted with appropriate caution and considered hypothesis-generating rather than confirmatory, as some apparent differences may reflect sampling error or technical artifacts rather than true biological variability. As a result, some apparent differences may reflect sampling error or technical artifacts rather than true biological variability.

For organs and metrics where no statistically significant differences were observed, it remains unclear whether this reflects genuine biological similarity or insufficient study power, for example due to limited sample size or cohort selection. The sample size difference between the male and female groups may also have contributed to the lack of statistical significance. Thus, the absence of statistical differences should not necessarily be equated with equivalence between groups.

The use of the Biograph Vision Quadra scanner represents a methodological strength, as its high sensitivity supports high-quality imaging at low doses, including imaging of small and challenging anatomical structures [8]. Organs were segmented on the low-dose CT using MOOSE, and random checks were performed to ensure anatomical and quantitative integrity; yet, the possibility of residual segmentation errors or individual anatomical variability cannot be fully excluded.

Future research should systematically address additional factors that may contribute to the observed differences, including body composition, hormonal influences including menopausal status, and evaluation of organs that could not be segmented in this study, in particular dopaminergic brain structures like the striatum. These efforts will facilitate a deeper understanding of sex- and age-related variability and promote a more individualized interpretation of PET images. Further studies are essential to elucidate the underlying causes of these differences and to further refine PET analysis protocols, taking into account both sex and organ-specific effects. Importantly, sex should be explicitly considered in all future PET research and clinical studies, given the significant differences identified in this study.

## 5. Conclusions

This study aimed to investigate sex- and age-related differences in the physiological distribution of [^18^F]FDOPA uptake on PET imaging across a range of organs and tissues. The results demonstrate that both sex and age contribute to organ- and metric-specific variability in quantitative PET parameters. While most organs exhibit comparable tracer uptake between males and females, males tend to show higher uptake in metabolically active organs, whereas females display greater intra-organ heterogeneity. aging is generally associated with increased PET tracer uptake and variability, particularly in women, with certain patterns, such as higher hip uptake in younger individuals highlighting the complexity of age-related metabolic change across tissues.

Importantly, statistically significant differences are not universal, and several factors, including hormonal status, body composition, technical methodology, and sample size may influence observed results. The strengths of this study include the use of high-sensitivity PET imaging and the normalization of uptake to lean body mass, which reduce bias and improve reliability. However, the limited sample sizes, and potential confounders such as unmeasured hormonal or metabolic status limit the generalizability of the findings.

These findings emphasize the need for careful consideration of both sex and age during PET image analysis and interpretation. Development and adoption of age- and sex-adjusted thresholds or reference values may improve the accuracy and personalization of quantitative PET imaging in both clinical and research settings. Future research should incorporate additional confounding variables and explore smaller or currently unsegmented organs to further refine personalized PET evaluation. 

## Figures and Tables

**Table 1:** Patient characteristics.

Patient Characteristics	N	%	
**Number of patients:**	106		
Male	48	45.3	
Age 18–50	18	17.0	
Age 51–90	30	28.3	
Female	58	54.7	
Age 18–50	28	26.4	
Age 51–90	30	28.3	
	**Male**	**Male 18–50**	**Male 51–90**
**Age (years)**			
Mean	54.3 ± 16.7	36.8 ± 12.3	64.8 ± 7.8
Range	50–83	19–50	51–83
**Weight (kg)**			
Mean	90.2 ± 15.3	92.6 ± 20.1	88.7 ± 11.8
**Height (cm)**			
Mean	181.3 ± 7.5	182.9 ± 6.9	180.2 ± 7.7
**LBM (kg)**			
Mean	65.9 ± 6.8	67.0 ± 7.8	65.2 ± 6.1
	**Female**	**Female 18–50**	**Female 51–90**
**Age (years)**			
Mean	49.7 ± 16.8	35.4 ± 10.2	63.2 ± 8.6
Range	18–77	18–50	51–77
**Weight (kg)**			
Mean	73.4 ± 14.8	69.6 ± 12.7	76.8 ± 15.9
**Height (cm)**			
Mean	168.9 ± 8.9	171.3 ± 8.0	166.6 ± 9.3
**LBM (kg)**			
Mean	44.8 ± 6.2	44.1 ± 5.5	45.5 ± 6.8

**Table 2:** Overview of excluded organs due to pathological reasons.

Number of Organs/Tissues Excluded	Reason for Exclusion
6× Adrenal gland Right	· Focal increased [^18^F]FDOPA uptake (2)
	· Increased [^18^F]FDOPA uptake (2)
	· Resection (1)
	· Not found in MOOSE (1)
5× Adrenal gland Left	· Focal increased [^18^F]FDOPA uptake (2)
	· Resection (1)
	· Enlarged (1)
	· Not found in MOOSE (1)
3× Hip	· Focal increased [^18^F]FDOPA uptake (1)
	· Total hip replacement left and right (1)
	· Total hip replacement right (1)
1× Brain	· Focal increased [^18^F]FDOPA uptake
1× Duodenum	· Resection
15× Gallbladder	· Increased [^18^F]FDOPA uptake (1)
	· Resection (8)
	· Not found in MOOSE (6)
2× Kidney Right	· Renal fusion (1)
	· Atrophic (1)
3× Kidney Left	· Focal increased [^18^F]FDOPA uptake (1)
	· Resection (1)
	· Renal fusion (1)
1× Left ventricle	· Increased [^18^F]FDOPA uptake
12× Liver	· Focal increased uptake (8)
	· Resection (2)
	· Transplantation (1)
	· Embolisations (1)
5× Pancreas	· Focal increased [^18^F]FDOPA uptake (1)
	· Increased [^18^F]FDOPA uptake (1)
	· Resection (2)
	· Partial resection (1)
1× Spleen	· Not found in MOOSE
4× Stomach	· Resection (2)
	· Intrathoracic stomach (1)
	· Not found in MOOSE (1)
6× Thyroid Right	· Focal increased [^18^F]FDOPA uptake (1)
	· Hemithyroidectomy (1)
	· Thyroidectomy (4)
4× Thyroid Left	· Thyroidectomy (4)

**Table 3:** Overview of the number of organs for statistical testing in sex comparison.

Case Processing Summary						
		Cases				
		Valid		Missing		Total
	Gender	N	Percent	N	Percent	N
Left ventricle	Male	48	100.0%	0	0.0%	48
	Female	57	98.3%	1	1.7%	58
Duodenum	Male	47	97.9%	1	2.1%	48
	Female	58	100.0%	0	0.0%	58
Adrenal gland left	Male	46	95.8%	2	4.2%	48
	Female	55	94.8%	3	5.2%	58
Adrenal gland right	Male	44	91.7%	4	8.3%	48
	Female	56	96.6%	2	3.4%	58
Brain	Male	48	100.0%	0	0.0%	48
	Female	57	98.3%	1	1.7%	58
Gallbladder	Male	42	87.5%	6	12.5%	48
	Female	50	86.2%	8	13.8%	58
Kidney left	Male	46	95.8%	2	4.2%	48
	Female	57	98.3%	1	1.7%	58
Kidney right	Male	45	93.8%	3	6.3%	48
	Female	58	100.0%	0	0.0%	58
Liver	Male	41	85.4%	7	14.6%	48
	Female	53	91.4%	5	8.6%	58
Pancreas	Male	45	93.8%	3	6.3%	48
	Female	56	96.6%	2	3.4%	58
Spleen	Male	47	97.9%	1	2.1%	48
	Female	58	100.0%	0	0.0%	58
Stomach	Male	48	100.0%	0	0.0%	48
	Female	54	93.1%	4	6.9%	58
Thyroid	Male	47	97.9%	1	2.1%	48
	Female	55	94.8%	3	5.2%	58
Hip	Male	46	95.8%	2	4.2%	48
	Female	57	98.3%	1	1.7%	58

**Table 4:** Results of normalcy test for each metric in sex comparison. Metrics are defined as: 1 = $SUL_{mean}$, 2 = $SUL_{max}$, 3 = TBR, and 4 = CoV.

	Parametric	Non-Parametric
left ventricle	3	1 2 4
duodenum	1 3	2 4
left adrenal gland	4	1 2 3
right adrenal gland	4	1 2 3
brain	1 2 3 4	
gallbladder		1 2 3 4
left kidney		1 2 3 4
right kidney		1 2 3 4
liver	3	1 2 4
pancreas	1	2 3 4
spleen		1 2 3 4
stomach	1 3	2 4
thyroid	1 3 4	2
hip	3 4	1 2

**Table 5:** Overview of the number of organs for statistical testing in female and male age comparison.

Case Processing Summary											
		Female Cases					Male Cases				
		Valid		Missing		Total	Valid		Missing		Total
	Agegroup	N	Percent	N	Percent	N	N	Percent	N	Percent	N
Left ventricle	Old	29	96.7%	1	3.3%	30	30	100.0%	0	0.0%	30
	Young	28	100.0%	0	0.0%	28	18	100.0%	0	0.0%	18
Duodenum	Old	30	100.0%	0	0.0%	30	29	96.7%	1	3.3%	30
	Young	28	100.0%	0	0.0%	28	18	100.0%	0	0.0%	18
Adrenal gland left	Old	29	96.7%	1	3.3%	30	30	100.0%	0	0.0%	30
	Young	26	92.9%	2	7.1%	28	16	88.9%	2	11.1%	18
Adrenal gland right	Old	30	100.0%	0	0.0%	30	28	93.3%	2	6.7%	30
	Young	26	92.9%	2	7.1%	28	16	88.9%	2	11.1%	18
Brain	Old	30	100.0%	0	0.0%	30	30	100.0%	0	0.0%	30
	Young	27	96.4%	1	3.6%	28	18	100.0%	0	0.0%	18
Gallbladder	Old	24	80.0%	6	20.0%	30	26	86.7%	4	13.3%	30
	Young	26	92.9%	2	7.1%	28	16	88.9%	2	11.1%	18
Kidney left	Old	30	100.0%	0	0.0%	30	29	96.7%	1	3.3%	30
	Young	27	96.4%	1	3.6%	28	17	94.4%	1	5.6%	18
Kidney right	Old	30	100.0%	0	0.0%	30	27	90.0%	3	10.0%	30
	Young	28	100.0%	0	0.0%	28	18	100.0%	0	0.0%	18
Liver	Old	28	93.3%	2	6.7%	30	26	86.7%	4	13.3%	30
	Young	25	89.3%	3	10.7%	28	15	83.3%	3	16.7%	18
Pancreas	Old	29	96.7%	1	3.3%	30	29	96.7%	1	3.3%	30
	Young	27	96.4%	1	3.6%	28	16	88.9%	2	11.1%	18
Spleen	Old	30	100.0%	0	0.0%	30	29	96.7%	1	3.3%	30
	Young	28	100.0%	0	0.0%	28	18	100.0%	0	0.0%	18
Stomach	Old	26	86.7%	4	13.3%	30	30	100.0%	0	0.0%	30
	Young	28	100.0%	0	0.0%	28	18	100.0%	0	0.0%	18
Thyroid	Old	29	96.7%	1	3.3%	30	30	100.0%	0	0.0%	30
	Young	26	92.9%	2	7.1%	28	17	94.4%	1	5.6%	18
Hip	Old	29	96.7%	1	3.3%	30	29	96.7%	1	3.3%	30
	Young	28	100.0%	0	0.0%	28	17	94.4%	1	5.6%	18

**Table 6:** Results of normalcy test for each metric. Metrics are defined as: 1 = $SUL_{mean}$, 2 = $SUL_{max}$, 3 = TBR, and 4 = CoV.

	Female		Male	
	Parametric	Non-Parametric	Parametric	Non-Parametric
left ventricle	3 4	1 2	1 2 3	4
duodenum	1 3	2 4	1	2 3 4
left adrenal gland	2	1 3 4	2 3 4	1
right adrenal gland		1 2 3 4	4	1 2 3
brain	2	1 3 4	1 2 3 4	
gallbladder		1 2 3 4	1 3	2 4
left kidney	3	1 2 4		1 2 3 4
right kidney	4	1 2 3	1 3	2 4
liver	1 3	2 4	1 3	2 4
pancreas	1 3 4	2	1 2	3 4
spleen		1 2 3 4	1 3	2 4
stomach	1 3	2 4	1 3	2 4
thyroid	1 2 3 4		1 3 4	2
hip	3	1 2 4	3 4	1 2

**Figure A1:**
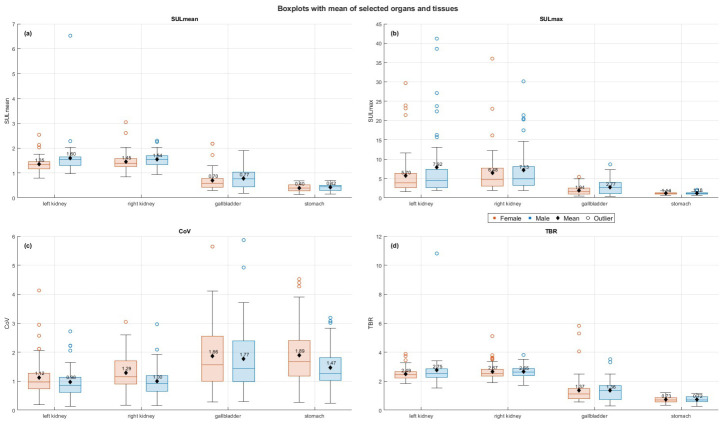
Boxplots with mean of the (**a**) $SUL_{mean}$, (**b**) $SUL_{max}$, (**c**) TBR and (**d**) CoV of selected organs in the sex comparison. Please note, the axes are intentionally scaled differently to improve visualization.

**Figure A2:**
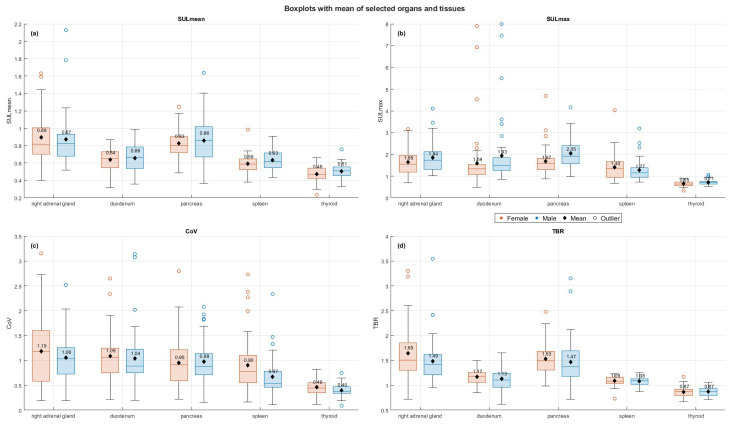
Boxplots with mean of the (**a**) $SUL_{mean}$, (**b**) $SUL_{max}$, (**c**) TBR and (**d**) CoV of selected organs in the sex comparison. Please note, the axes are intentionally scaled differently to improve visualization.

**Figure A3:**
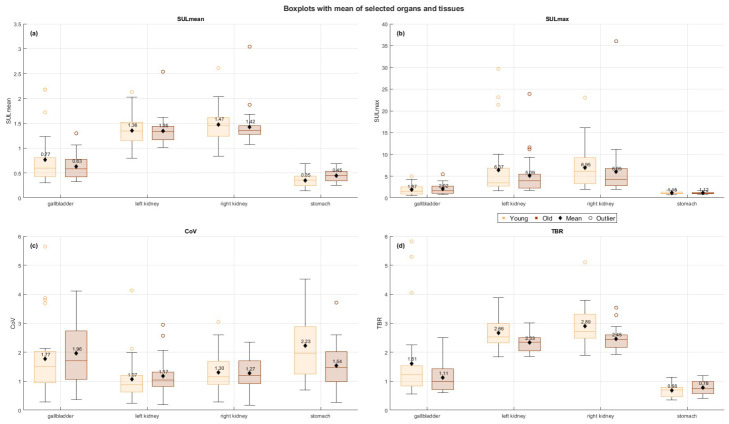
Boxplots with mean of the (**a**) $SUL_{mean}$, (**b**) $SUL_{max}$, (**c**) TBR and (**d**) CoV of selected organs in the age comparison in females. Please note, the axes are intentionally scaled differently to improve visualization.

**Figure A4:**
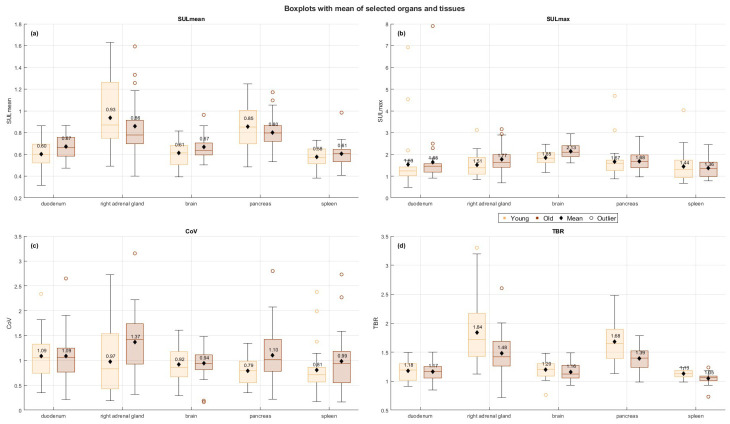
Boxplots with mean of the (**a**) $SUL_{mean}$, (**b**) $SUL_{max}$, (**c**) TBR and (**d**) CoV of selected organs in the age comparison in females. Please note, the axes are intentionally scaled differently to improve visualization.

**Figure A5:**
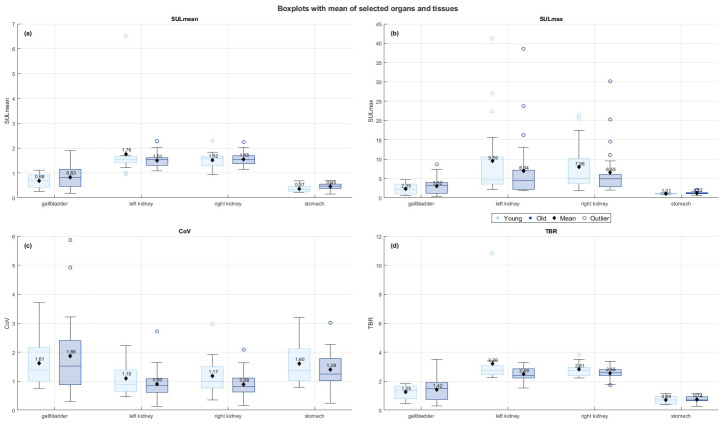
Boxplots with mean of the (**a**) $SUL_{mean}$, (**b**) $SUL_{max}$, (**c**) TBR and (**d**) CoV of selected organs in the age comparison in males.Please note, the axes are intentionally scaled differently to improve visualization.

**Figure A6:**
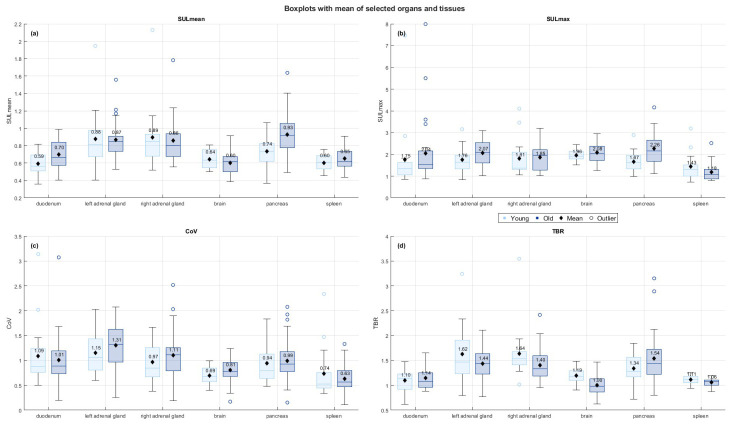
Boxplots with mean of the (**a**) $SUL_{mean}$, (**b**) $SUL_{max}$, (**c**) TBR and (**d**) CoV of selected organs in the age comparison in males. Please note, the axes are intentionally scaled differently to improve visualization.

## Data Availability

The data presented in this study are not publicly available due to privacy and ethical restrictions.

## Abbreviations

The following abbreviations are used in this manuscript:

CoV	Coefficient of variation
CT	Computed tomography
LBM	Lean body mass
PET	Positron emission tomography
SULmax	Maximum standarized uptake value normalized for lean body mass
SULmean	Mean standarized uptake value normalized for lean body mass
TBR	Target-to-background ratio

## Appendix A

**Table A1:** Test statistics of sex differences for each organ or tissue. Abbreviations are defined as: MWU = Mann-Whitney U test, IST = Independent samples *t*-test. In case of the Mann-Whitney U test the Z-value and 2-tailed *p*-value is given. In case of the independent samples *t*-test the t(df), with df = degrees of freedom and the 2-tailed *p*-value are given. * significant difference.

	SULmean			SULmax			TBR			CoV		
Organ or Tissue	Test	*p*-Value	t(df) or Z	Test	*p*-Value	t(df) or Z	Test	*p*-Value	t(df) or Z	Test	*p*-Value	t(df) or Z
left ventricle	MWU	0.148	−1.447	MWU	0.007 *	−2.721	IST	0.870	−0.164(103)	MWU	0.002 *	−3.094
duodenum	IST	0.473	0.721(103)	MWU	0.023 *	−2.275	IST	0.246	−1.166(103)	MWU	0.241	−1.173
left adrenal gland	MWU	0.079	−1.759	MWU	0.034 *	−2.121	MWU	0.003 *	−2.987	IST	0.581	0.554(95.5)
right adrenal gland	MWU	0.723	−0.354	MWU	0.197	−1.292	MWU	0.080	−1.750	MWU	0.359	−0.917
brain	IST	0.328	−0.983(103)	IST	0.644	0.463(103)	IST	0.003 *	−3.075(103)	IST	0.002 *	−3.177(101.0)
gallbladder	MWU	0.173	−1.364	MWU	0.061	−1.873	MWU	0.335	−0.964	MWU	0.610	-0.510
left kidney	MWU	0.001 *	−3.430	MWU	0.187	−1.320	MWU	0.158	−1.413	MWU	0.176	−1.353
right kidney	MWU	0.027 *	−2.214	MWU	0.894	−0.133	MWU	0.523	−0.638	MWU	0.007 *	−2.719
liver	MWU	0.003 *	−2.924	MWU	0.001 *	−3.244	IST	0.041 *	2.071(92)	MWU	0.510	−0.660
pancreas	IST	0.452	0.757(76.1)	MWU	0.002 *	−3.123	MWU	0.142	−1.469	MWU	0.774	−0.287
spleen	MWU	0.093	−1.682	MWU	0.195	−1.295	MWU	0.782	−0.277	MWU	0.004 *	−2.874
stomach	IST	0.411	0.826(100)	MWU	0.252	−1.146	IST	0.911	−0.112(100)	MWU	0.035 *	−2.112
thyroid	IST	0.078	1.782(100)	MWU	0.008 *	-2.655	IST	0.876	0.156(100)	IST	0.052	−1.971(97.8)
hip	MWU	0.958	−0.053	MWU	0.014 *	−2.461	IST	0.085	−1.740(101)	IST	0.015 *	−2.477(101.0)

**Table A2:** Test statistics of gender differences in females for each organ or tissue. Abreviations are defined as: MWU = Mann-Whitney U test, IST = Independent samples *t*-test. In case of the Mann-Whitney U test the Z-value and 2-tailed *p*-value is given. In case of the independent samples *t*-test the t(df), with df = degrees of freedom and the 2-tailed *p*-value are given. * significant difference.

	SULmean			SULmax			TBR			CoV		
Organ or Tissue	Test	*p*-Value	t(df) or Z	Test	*p*-Value	t(df) or Z	Test	*p*-Value	t(df) or Z	Test	*p*-Value	t(df) or Z
left ventricle	MWU	0.000 *	−4.358	MWU	0.000 *	−4.996	IST	0.000 *	3.806(55)	IST	0.669	0.430(55)
duodenum	IST	0.031 *	2.218(56)	MWU	0.060	−1.883	IST	0.810	−0.242(56)	MWU	0.950	−0.062
left adrenal gland	MWU	0.021 *	−2.310	IST	0.018 *	2.443(53)	MWU	0.000 *	−3.911	MWU	0.001 *	−3.287
right adrenal gland	MWU	0.278	−1.084	MWU	0.104	−1.626	MWU	0.014 *	−2.464	MWU	0.019 *	−2.349
brain	MWU	0.093	−1.678	IST	0.001 *	3.379(55)	MWU	0.218	−1.231	MWU	0.533	−0.623
gallbladder	MWU	0.771	−0.291	MWU	0.461	−0.738	MWU	0.268	−1.107	MWU	0.382	−0.874
left kidney	MWU	0.898	−0.128	MWU	0.911	−0.112	IST	0.006 *	−2.879(42.9)	MWU	0.129	−1.518
right kidney	MWU	0.297	−1.043	MWU	0.202	−1.276	MWU	0.002 *	−3.128	IST	0.861	−0.176(56)
liver	IST	0.021 *	2.372(51)	MWU	0.695	−0.392	IST	0.934	0.084(52)	MWU	0.028 *	−2.192
pancreas	IST	0.257	−1.146(54)	MWU	0.248	−1.156	IST	0.000 *	−3.886(42.8)	IST	0.015 *	2.536(43.2)
spleen	MWU	0.359	−0.918	MWU	0.950	−0.062	MWU	0.001 *	−3.330	MWU	0.135	−1.494
stomach	IST	0.010 *	2.693(52)	MWU	0.863	−0.173	IST	0.134	1.524(52)	MWU	0.030 *	−2.164
thyroid	IST	0.004 *	3.041(53)	IST	0.006 *	2.849(53)	IST	0.306	1.033(53)	IST	0.365	0.914(53)
hip	MWU	0.032 *	−2.139	MWU	0.000 *	−3.991	IST	0.000 *	−6.510(55)	MWU	0.114	−1.580

**Table A3:** Test statistics of gender differences in males for each organ or tissue. Abreviations are defined as: MWU = Mann-Whitney U test, IST = Independent samples *t*-test. In case of the Mann-Whitney U test the Z-value and 2-tailed *p*-value is given. In case of the independent samples *t*-test the t(df), with df = degrees of freedom and the 2-tailed *p*-value are given. * significant difference.

	SULmean			SULmax			TBR			CoV		
Organ or Tissue	Test	*p*-Value	t(df) or Z	Test	*p*-Value	t(df) or Z	Test	*p*-Value	t(df) or Z	Test	*p*-Value	t(df) or Z
left ventricle	IST	0.001 *	3.590(44.2)	IST	0.002 *	3.328(46,0)	IST	0.105	1.653(47)	MWU	0.848	−0.192
duodenum	IST	0.025 *	2.327(45)	MWU	0.092	−1.685	MWU	0.975	−0.032	MWU	0.930	−0.088
left adrenal gland	MWU	0.747	−0.323	IST	0.084	1.766(44)	IST	0.191	−1.350(21.8)	IST	0.267	1.124(44)
right adrenal gland	MWU	0.807	−0.244	MWU	0.626	−0.488	MWU	0.044 *	−2.013	IST	0.361	0.924(42)
brain	IST	0.236	−1.201	IST	0.301	1.047(46)	IST	0.001 *	−3.718(47)	IST	0.085	1.759(46)
gallbladder	IST	0.234	1.209(40)	MWU	0.379	−0.881	IST	0.577	0.562(41)	MWU	0.717	−0.363
left kidney	MWU	0.562	−0.580	MWU	0.369	−0.899	MWU	0.051	−1.948	MWU	0.381	−0.876
right kidney	IST	0.723	0.357(43)	MWU	0.404	−0.834	IST	0.056	−1.965(44)	MWU	0.138	−1.483
liver	IST	0.007 *	2.845(39)	MWU	0.516	−0.650	IST	0.271	1.117(40)	MWU	0.978	−0.027
pancreas	IST	0.012 *	2.623(43)	IST	0.004 *	3.017(43)	MWU	0.357	−0.922	MWU	0.407	−0.830
spleen	IST	0.162	1.421(45)	MWU	0.076	−1.773	IST	0.024 *	−2.331(46)	MWU	0.913	−0.109
stomach	IST	0.046 *	2.048(46)	MWU	0.058	−1.895	IST	0.744	0.329(47)	MWU	0.609	−0.511
thyroid	IST	0.017 *	2.485(45)	MWU	0.127	−1.528	IST	0.750	0.320(46)	IST	0.925	0.095(45)
hip	MWU	0.016 *	−2.401	MWU	0.794	−0.262	IST	0.000 *	−7.143(45)	IST	0.420	0.813(44)
